# The Association of Sugar-Sweetened Beverages Consumption Patterns and Overweight/Obesity: Evidence from a Large-Scale Survey of Chinese Children and Adolescents

**DOI:** 10.3390/nu17213442

**Published:** 2025-10-31

**Authors:** Yi Liu, Feng Pan, Jin-Lang Lyu, Jian-Wen Li, Jiao Xu, Hai-Jun Wang, Dong Liang

**Affiliations:** 1 Department of Maternal and Child Health, School of Public Health, Peking University, National Health Commission Key Laboratory of Reproductive Health, Beijing 100191, China; liuyi627@bjmu.edu.cn (Y.L.); jinlanglyu@bjmu.edu.cn (J.-L.L.); 2 China National Center for Food Safety Risk Assessment, Beijing 100022, China; panfeng@cfsa.net.cn (F.P.); lijianwen@cfsa.net.cn (J.-W.L.)

**Keywords:** sugar-sweetened beverages, children and adolescent, obesity, consumption pattern, China

## Abstract

**Objective:** To identify major sugar-sweetened beverage (SSB) consumption patterns among Chinese children and adolescents and examine their associations with childhood overweight/obesity. **Methods:** Data were drawn from the Chinese Food Consumption Survey (2017–2020), including 7979 children and adolescents. SSB intake was assessed using a non-consecutive 3-day 24 h dietary recall and classified into nine types. Principal component analysis identified SSB consumption patterns. Nutritional status was defined using BMI Z-scores, following the World Health Organization growth standards. Multivariable logistic regression was used to assess the associations between SSB patterns and overweight/obesity, with subgroup analyses by sex, age, and residence area. **Results:** Three major SSB patterns were identified: (1) Carbonated Beverage and Milk Tea Pattern (dominated by carbonated beverages and milk tea); (2) Functional Beverages Pattern (dominated by coffee beverages and sports beverages); and (3) Plant Hybrid Pattern (dominated by plant protein beverages and plant-based beverages). Preschool-aged children exhibited lower scores across all three patterns. Higher pattern scores were observed among school-aged children and adolescents and those with lower parental education levels, parents working as unskilled labor or homemakers, lower family annual income per capita, and residence in rural areas. All three identified SSB consumption patterns demonstrated significant positive associations with overweight/obesity in children, where higher consumption levels corresponded to greater odds of overweight/obesity. Children exhibiting higher scores in two or more patterns had higher odds of being overweight/obese (Medium-high: OR = 1.249, 95% CI = 1.053, 1.482; High: OR = 1.256, 95% CI = 1.081, 1.459). Subgroup analysis further indicated that the association between the Plant Hybrid Pattern score and overweight/obesity varied significantly by sex. **Conclusions:** Three SSB consumption patterns were associated with a higher likelihood of overweight/obesity among Chinese children, particularly among those with preferences for multiple SSB types. Interventions should be tailored to SSB consumption habits and socioeconomic contexts, with special attention to rural populations.

## 1. Introduction

Childhood overweight/obesity has become a major global public health concern, with its prevalence rising over recent decades. Over 390 million children and adolescents aged 5–19 were overweight in 2022, with 160 million suffering with obesity [1]. The total number of overweight children under the age of 5 reached 35.5 million in 2024 [2]. The prevalence of childhood overweight/obesity in China was 19% among children and adolescents aged 6–17 and 10.4% among those under 6 years old, respectively, according to data from the China Chronic Disease and Nutrition Surveillance Study (2015–2019) [3]. While childhood obesity remained prevalent in urban areas, recent evidence suggested narrowing urban–rural disparities, particularly in less developed regions. National surveillance data from 1985 to 2019 showed consistently higher obesity rates among urban children; however, by 2019, obesity prevalence among rural girls in provinces with the most advantaged socioeconomic status (SES) had surpassed that of their urban counterparts (urban–rural gap: −0.37%, 95% CI: −0.07, −0.80) [4]. It underscored the shifting burden of childhood overweight/obesity toward socioeconomically disadvantaged populations, especially in rural areas.

From 1990 to 2018, the prevalence of sugar-sweetened beverage (SSB) intake among children and adolescents in 185 countries rose by 23% [5]. SSBs have well-documented contributions to childhood overweight/obesity in high-income, low-income, and middle-income countries [6]. Evidence also pointed to significant variation in SSB consumption by demographic characteristics. In China, a national survey reported that 14.8% of adolescents consumed SSBs more than four times per week [7]. For preschoolers, 84.5% of children consumed SSBs in the past three months [8]. Additionally, boys tended to consume more SSBs than girls, and differences existed across age groups and geographic regions [9]. Moreover, socioeconomic factors, such as parental education and household income, were strongly associated with children’s dietary habits, including SSB consumption [6,10,11].

Importantly, beyond the total amount of SSBs consumed, the types and patterns of consumption may also influence obesity risk, yet previous studies have largely focused on quantity rather than beverage variety [12]. In China, the market for SSBs is highly diverse, and children and adolescents exhibited varied consumption preferences. For example, preschoolers most frequently consumed milk beverages, followed by fruit and vegetable beverages and plant-based protein drinks, whereas adolescents tend to prefer tea beverages and carbonated soft drinks [8,13]. However, all of above studies were conducted in a specific region. Different types of SSBs may have varying associations with childhood overweight/obesity [14]. Notably, in recent years, the increasing popularity of milk tea and other special beverages may have altered children’s beverage consumption patterns. Nevertheless, most previous studies focused on individual beverage types or overall consumption volume, rather than examining patterns of SSB consumption. Although some researchers have investigated SSB consumption patterns among children in certain regions of China, nationwide data remains limited [15]. Understanding SSB consumption patterns is crucial for identifying high-risk groups and for designing more targeted and effective public health interventions related to SSB intake and choice, especially when considering evolving preferences and trends.

Therefore, this study aimed to (1) identify major SSB consumption patterns among Chinese children and adolescents; (2) assess the relationships between different SSB consumption patterns and childhood overweight/obesity; and (3) investigate whether these associations vary across subgroups classified by sex, age, and residence area.

## 2. Methods

### 2.1. Study Design and Participants

The data for this study were sourced from the Chinese Food Consumption Survey (CFCS), conducted annually from 2017 to 2020. The CFCS is a nationally representative cross-sectional study to evaluate food consumption patterns, dietary nutrient intakes, and potential food safety risks among the Chinese population, conducted by the China National Center for Food Safety Risk Assessment. This survey employed a multi-stage stratified cluster random sampling approach to systematically collect representative sample data in 23 provinces (or autonomous regions and municipalities) across China. All household members aged 3 years and older were eligible and included in the survey. This study included children and adolescents aged 3 to 17 years with complete survey data. The CFCS received approval from the Ethics Committee of the China National Center for Food Safety Risk Assessment (CFSA). Informed consent was obtained from the parents or legal guardians of all children and adolescents (under 18 years) before participation in the survey.

To evaluate whether the available sample size had sufficient statistical power under the expected effect size, a hypothesis-testing based formula was applied: $N=\;\frac{{\left({Ζ}_{1-\frac{\alpha }{2}}+{Ζ}_{1-\beta }\right)}^{2}\times \;[p_{1}(1-p_{1})+p_{2}(1-p_{2})]}{{\left(p_{1}-p_{2}\right)}^{2}}$, and the parameters were set as follows: *N* denotes the required sample size per group; ${Ζ}_{1-\frac{\alpha }{2}}\;$ represents the standard normal deviate for a two-sided test at a significance level of α = 0.05 (Z ≈ 1.96); ${Ζ}_{1-\beta }$ is the deviate corresponding to 80% power (β = 0.20, Z ≈ 0.84); $p_{1}$ is the outcome prevalence in the unexposed group; and $p_{2}$ is the outcome prevalence in the exposed group. The $p_{2}$ was derived from the odds ratio (OR) using the following formula: $p_{2}=\;\frac{OR\times p_{1}}{1-p_{1}+OR\times p_{1}}$. Based on data from the China Chronic Disease and Nutrition Surveillance survey (2015–2019), the prevalence of overweight and obesity among children and adolescents aged 6–17 years was 19%, and the prevalence among those under 6 years was 10.4% [3]. Considering the overall prevalence of overweight/obesity in our study population (30.58%) and an anticipated minimum detectable OR of 1.55 between the highest and lowest SSB intake groups [16], the minimum required total sample size was estimated at 1672 participants, assuming a 2:1 ratio of control to case groups and 80% statistical power, corresponding to 1115 controls and 557 cases. A total of 7979 children and adolescents with normal weight and overweight/obesity were included in the final analysis. In addition, the sample size was sufficient to support further stratified analyses involving up to three subgroups.

### 2.2. SSB Consumption Measurement and Patterns Construction

The consumption of SSBs was evaluated by a non-consecutive 3-day 24 h dietary recall method to estimate the daily varieties and quantities of SSB intake among children and adolescents. Each participant completed three separate 24 h dietary recalls, collected through face-to-face, household-based questionnaire interviews, collecting detailed information on the intake of all foods and beverages (including drinking water) within the 24 h prior to each interview. The three recalls comprised two weekdays and one weekend day (Saturday or Sunday) and were conducted with a minimum interval of one full day between each. For children below 12 years of age, dietary recalls were conducted with the assistance of parents or primary caregivers. According to the General Standard for Beverages (GB/T 10789-2015) [17], SSBs were categorized into nine groups: carbonated beverages, fruit and vegetable beverages, plant protein beverages, dairy-containing beverages, tea beverages, coffee beverages, plant-based beverages, sports beverages, and milk tea.

Principal component factor analysis was utilized to identify patterns of SSB consumption. Prior to the analysis, pairwise correlations among the different SSB categories were examined. The overall adequacy of the data was further validated by the Kaiser–Meyer–Olkin (KMO) test and Bartlett’s test of sphericity (*p* < 0.001). The number of factors to retain was determined according to eigenvalues above 1, scree plots, parallel analysis, and the interpretability of the factors [18]. Components of the SSB consumption patterns were identified by absolute factor loadings over 0.5.

### 2.3. The Criteria for Diagnosing Childhood Overweight/Obesity

Anthropometric measurements were collected by trained investigators following standardized measurement procedures and equipment guidance during household visits. Body weight and height were reported to the nearest 0.1 kg and 0.1 cm, respectively. The body mass index (BMI) was computed by dividing weight by the square of height (kg/m^2^). Nutritional status was evaluated using age- and sex-specific BMI Z-scores in accordance with the World Health Organization (WHO) growth standards [19,20]. For children under 5 years of age, overweight was defined as a BMI Z-score > +2 standard deviations (SD), whereas obesity was defined as a BMI Z-score > +3 SD. For children and adolescents aged 5–17 years, overweight was characterized by a BMI Z-score > +1 SD, whereas obesity was indicated by a BMI Z-score > +2 SD. Children with an absolute value of BMI Z-score ≥ 5 were excluded from this study. In line with the study objectives, underweight children were also excluded, and only those with normal weight or overweight/obesity were included in the final analysis.

### 2.4. Covariates

Demographic information included age, sex, area of residence, parental educational qualification and occupation, and annual household income per capita. The data was collected via face-to-face household interviews. Areas were classified into large cities, small and medium-sized cities, and rural regions according to the economic development level. Large cities were defined as the main urban areas of cities with a permanent resident urban population of more than one million. Medium and small cities referred to the main urban areas of cities with a resident urban population of less than one million. Rural survey sites included county-level administrative units in China, such as counties, autonomous counties, banners, autonomous banners, special regions, or forestry areas [21]. Parental educational qualification was classified according to years of completed education as none or primary, secondary, and trade school/college/university [22]. Parental occupation was categorized into unskilled workers or homemakers, skilled workers, and professionals/managers [22]. Parental height and weight were measured by trained investigators utilizing standardized methods, and BMI was computed accordingly. Parental nutritional status was defined as underweight (BMI < 18.5 kg/m^2^), normal weight (18.5 ≤ BMI < 24 kg/m^2^), overweight (24 ≤ BMI < 28 kg/m^2^), and obesity (BMI ≥ 28 kg/m^2^) [23]. Children’s total daily energy intake and protein energy ratio were assessed using a non-consecutive 3-day 24 h dietary recall method.

### 2.5. Statistical Analysis

Categorical variables were presented as counts (percentages). Continuous variables were described as mean ± SD for normally distributed data and as median with interquartile range (IQR) for skewed distributions. The chi-square test was used to compare categorical variables across groups. Multivariable logistic regression models were applied to investigate the relationship between SSB consumption patterns and overweight/obesity in children. Two models were created for each analysis: Model 1 adjusted for basic demographic variables (including age in years, sex, residential area, parental educational qualification, and family annual income per capita), and Model 2 further controlled for the quantity of SSB intake (mL/day).

For each identified major pattern, participants in the lowest tertile were categorized as the “Low” group, while those in the upper two tertiles were categorized as the “High” group. In addition, a new variable, termed the “composite SSBs score”, was also established to reflect children’s exposure to multiple SSB patterns. Specifically, participants whose scores were “Low” across all three patterns were classified as Group 1. Those with “High” scores on exactly one pattern were classified as Group 2; those with “High” scores on two patterns were classified as Group 3; and those with “High” scores on three patterns were classified as Group 4. The relationship between the new variable and childhood overweight/obesity was also analyzed, and a trend test across the four groups was also conducted.

To further analyze potential heterogeneity in the relationship between SSB consumption patterns and overweight/obesity among subgroups, stratified analyses were conducted by sex (boys and girls), age groups (preschool children: 3–6 years, school-age children: 7–11 years, and adolescents: 12–17 years), and residential area (large cities, small and medium-sized cities, and rural areas). To examine whether there were significant interactions between SSB consumption patterns and age, sex, and area, we conducted likelihood ratio tests (LRTs) by comparing models with and without the interaction terms. To reduce the likelihood of false positives in the subgroup interaction analyses, *p*-values were corrected for multiple comparisons using the false discovery rate (FDR) method.

Sensitivity analysis was also conducted to test the robustness of our results by separately evaluating the associations between each SSB consumption pattern and the outcomes of childhood overweight and obesity separately. All data management and statistical analyses were performed utilizing R version 4.4.0. Two-tailed tests were conducted. A *p*-value < 0.05 was deemed statistically significant, and 95% confidence intervals (CIs) were reported.

## 3. Results

### 3.1. Participants Characteristics

**Figure 1:**
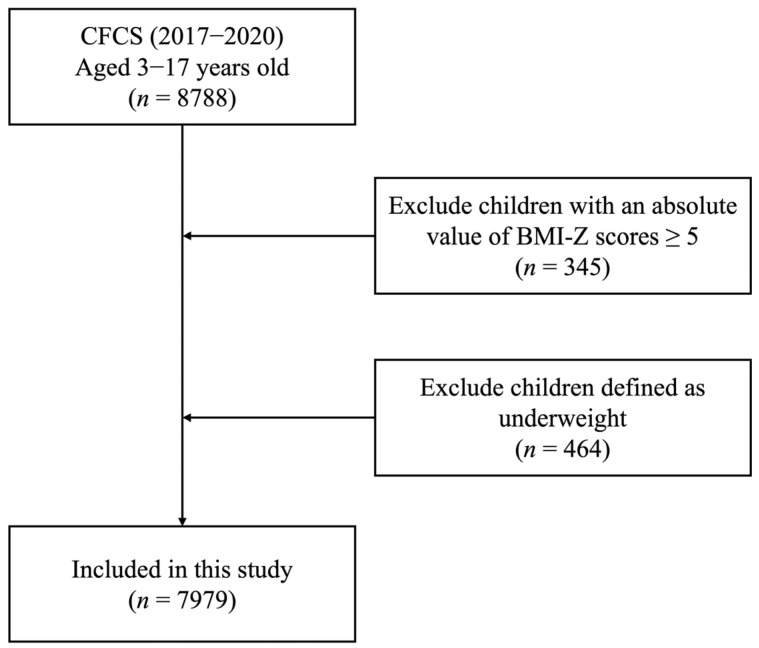
Flowchart of the participant selection process for the study.

A flowchart of the selection process for eligible participants is shown in Figure 1. This study included a total sample of 7979 children, of whom 5397 had normal weight, 1585 were with overweight, and 997 were with obesity. Among the total participants, 39.6% were school-aged children, 52.2% were male, and 39.4% resided in rural area. The majority of their parents had secondary education or were unskilled workers or homemakers. Additionally, 38.31% of families had an annual income per capita ranging from 10,000 to 20,000 (Table 1).

### 3.2. SSB Consumption Patterns Identification and Characteristics of Study Participants

**Figure 2:**
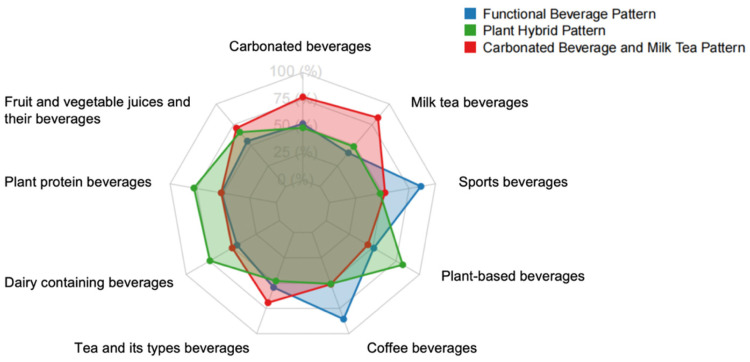
Radar chart of SSB consumption patterns derived from factor analysis.

The SSB consumption patterns identified through principal component analysis are presented (Figure 2, Supplementary Table S1). Factor analysis revealed three major SSB consumption patterns derived from nine beverage types, accounting for 12.07% (pattern 1: Carbonated Beverage and Milk Tea Pattern), 12.18% (pattern 2: Functional Beverage Pattern), and 11.78% (pattern 3: Plant Hybrid Pattern) of the variance, respectively, and explaining a total of 36.03% of the variance. The Carbonated Beverage and Milk Tea Pattern was dominated by carbonated beverages and milk tea beverages. The Functional Beverage Pattern was primarily characterized by high consumption of coffee beverages and sports beverages. The Plant Hybrid Pattern was defined by higher intake of plant protein beverages and plant-based beverages.

**Figure 3:**
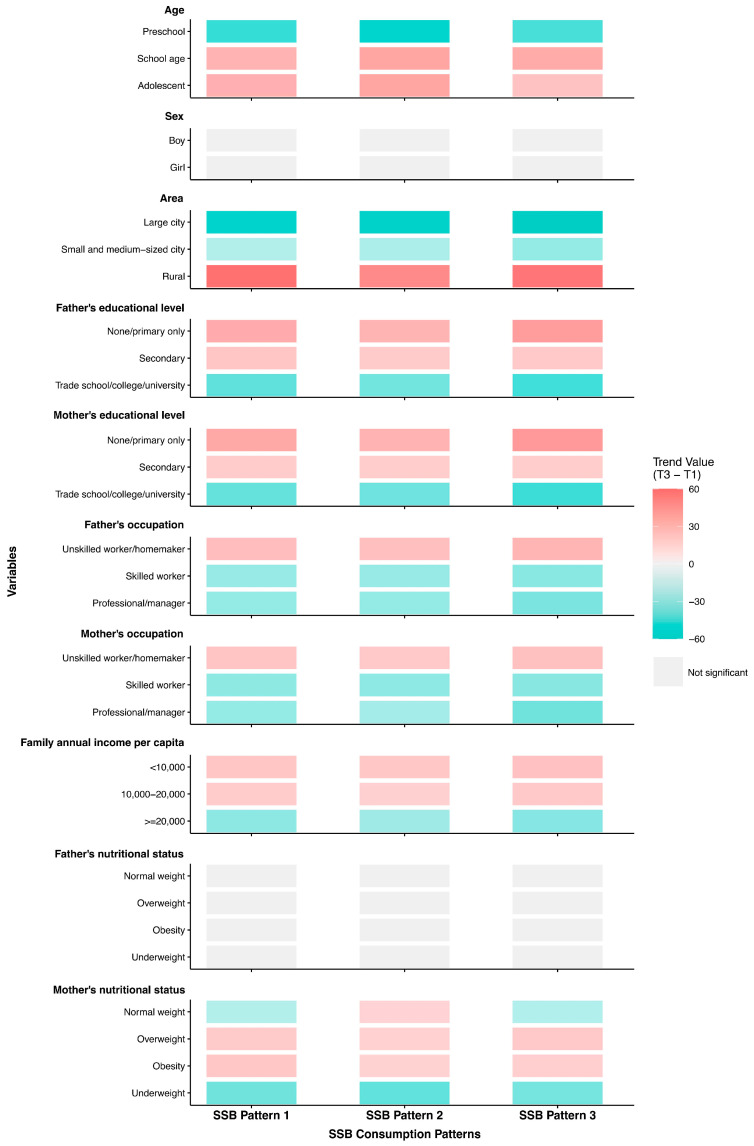
Summary of SSB consumption patterns by participant characteristics. SSB pattern 1 stood for Carbonated Beverage and Milk Tea Pattern; SSB pattern 2 stood for Functional Beverage Pattern; SSB pattern 3 stood for Plant Hybrid Pattern. The color intensity in the chart represented the magnitude of the difference, with darker shades indicating greater difference.

We described the attributes of children in the T1 (lowest) and T3 (highest) quartiles for the three SSB consumption patterns (Figure 3, Table 2, Supplementary Table S2) and composite SSBs score (Supplementary Table S3). Children of preschool age showed lower scores across all three SSB patterns. Children whose parents held no education or primary school education and whose occupations were unskilled workers or homemakers, who belonged to the lowest family annual income per capita group, and who resided in rural areas, tended to achieve high scores across all three patterns. Adolescents and those whose mothers were overweight or obese exhibited a greater propensity to conform to the Carbonated Beverage and Milk Tea Pattern. It was more probable that adolescents and whose mothers were obese would fit the Functional Beverage Pattern. Children in the top tertile of the Plant Hybrid Pattern were more likely to be of school age and adolescent and had mothers with overweight or obesity.

### 3.3. The Association Between SSB Consumption Patterns and Childhood Overweight/Obesity

We illustrated the correlation between three SSB consumption patterns and childhood overweight/obesity (Table 3). Upon adjusting for basic covariates (age in years, sex, region, parental education level, mother’s BMI, protein energy ratio, family annual income per capita) and household clustering as a random effect term in Model 1, significant results were found between high scores of Carbonated Beverage and Milk Tea Pattern (OR = 1.151, 95% CI = 1.010, 1.312), Functional Beverage Pattern (OR = 1.216, 95% CI = 1.071, 1.381), and Plant Hybrid Pattern (OR = 1.164, 95% CI = 1.028, 1.320) and childhood overweight/obesity. With additional adjustment for the quantity of SSB intake, the significant results remained (Carbonated Beverage and Milk Tea Pattern: OR = 1.158, 95% CI = 1.015, 1.321; Functional Beverage Pattern: OR = 1.216, 95% CI = 1.071, 1.381; Plant Hybrid Pattern: OR = 1.162, 95% CI = 1.026, 1.317).

Two pattern scores being high or all three pattern scores being high was both associated with higher likelihood of childhood overweight/obesity in Model 1 (Medium-high: OR = 1.236, 95% CI = 1.043, 1.465; High: OR = 1.253, 95% CI = 1.079, 1.456) and Model 2 (Medium-high: OR = 1.249, 95% CI = 1.053, 1.482; High: OR = 1.256, 95% CI = 1.081, 1.459), in comparison to children with low consumption across all three patterns (Table 3). The trend tests were significant in both Model 1 (*p* for trend = 0.004) and Model 2 (*p* for trend = 0.003).

### 3.4. Subgroup Analysis of SSB Consumption Patterns and Childhood Overweight/Obesity Across Different Age, Sex, and Area

**Figure 4:**
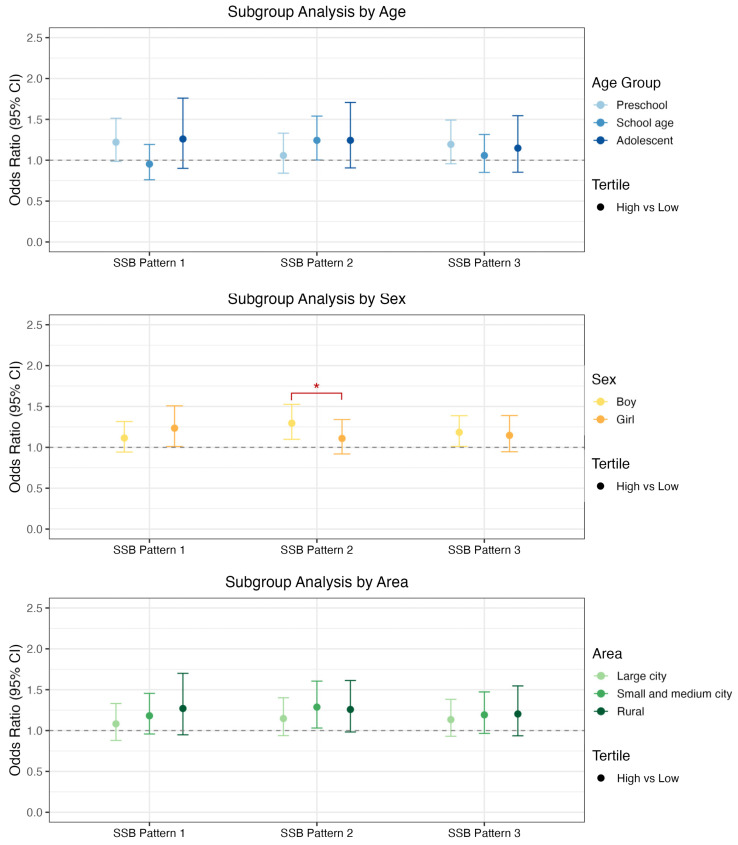
Forest plots for the association between SSB consumption patterns score and childhood overweight/obesity in subgroup analysis. SSB pattern 1 stands for Carbonated Beverage and Milk Tea Pattern; SSB pattern 2 stands for Functional Beverage Pattern; SSB pattern 3 stands for Plant Hybrid Pattern. * indicates a significant difference between subgroups at *p* < 0.05.

When stratified by age group, no significant effects were observed for all three patterns except for the Functional Beverage Pattern and children of school age (OR = 1.243, 95% CI = 1.003, 1.540) (Figure 4, Supplementary Table S6). In examining the significant effects associated with childhood overweight/obesity stratified by sex, a high score of the Carbonated Beverage and Milk Tea Pattern was associated with greater odds of childhood overweight/obesity compared with low score among girls (OR = 1.234, 95% CI = 1.010, 1.508). High score of the Functional Beverage Pattern (OR = 1.295, 95% CI = 1.098, 1.526) and Plant Hybrid Pattern (OR = 1.184, 95% CI = 1.011, 1.387) were associated with greater odds of childhood overweight/obesity compared with low score among boys (Supplementary Table S7). The interaction analysis revealed that the interaction between SSB pattern 2 and sex demonstrated a statistically significant effect (*p* = 0.005). After FDR correction, this interaction remained statistically significant (FDR-adjusted *p* = 0.015). When stratified with different areas, it showed a considerable threat to the Functional Beverage Pattern’s high score both in small and medium-sized cities (OR = 1.287, 95% CI = 1.031, 1.605) (Supplementary Table S8), while the *p* for interaction was not significant (Supplementary Table S8).

### 3.5. Sensitivity Analysis

We also examined the associations of the three SSB consumption patterns with overweight and obesity outcomes separately (Supplementary Tables S4 and S5). After adjusting for all covariates, high score of the Carbonated Beverage and Milk Tea Pattern was significantly associated with overweight (OR = 1.167, 95% CI = 1.001, 1.361), while high score of the Functional Beverage Pattern (OR = 2.328, 95% CI = 1.139, 4.760) was significantly associated with obesity. Having two pattern scores in the high category or all three pattern scores in the high category was significantly associated with overweight (Medium-high: OR = 1.252, 95% CI = 1.026, 1.528; High: OR = 1.208, 95% CI = 1.014, 1.439), while no significant association was found among children with obesity. The trend tests for both overweight (*p* for trend = 0.034) and obesity (*p* for trend = 0.042) models were statistically significant.

## 4. Discussion

In this study, we recognized three major SSB consumption patterns among Chinese children and adolescents. When comparing children in the highest and lowest consumption quartiles, those with higher SSB pattern scores were more likely to be of school age or adolescence, living in rural areas, and from families with parental education as none or primary, parental occupation as unskilled workers or homemakers, parental overweight/obesity, and the lowest annual household income per capita. Higher scores across all three SSB patterns were consistently associated with higher likelihood of overweight/obesity. When the three SSB patterns were composite into a single composite variable, children with medium-high and high consumption similarly exhibited greater odds of overweight/obesity. Subgroup analyses stratified by sociodemographic factors (age, sex, and area) indicated heterogeneity across subgroups, while a statistically significant interaction was identified exclusively between SSB consumption pattern 2 and sex.

Three main SSB consumption patterns were identified among Chinese children and adolescents: the Carbonated Beverage and Milk Tea Pattern, the Functional Beverage Pattern, and the Plant Hybrid Pattern. We applied principal component analysis, a method commonly used to identify dietary patterns in previous research and increasingly employed to explore beverage consumption patterns as well [24,25]. For example, a cross-sectional study conducted among 9–17-year-old children in Guangzhou also identified three SSB consumption patterns, the Plant Protein Pattern, Dairy-Containing Pattern, and Coffee Pattern, and found that all three patterns were associated with higher likelihood of undernutrition, after adjusting for dietary preferences and nutrition knowledge [15]. While the previous study was limited to regional samples from Guangdong and did not include younger (including preschool-aged) children, in our study we included a broader age range of children and adolescents using a nationwide representative sample. This approach enabled us to capture a more comprehensive understanding of SSB consumption patterns and preferences among Chinese children and adolescents. The findings suggest that SSB consumption behaviors could be an important component of childhood obesity prevention strategies, with substantial implications for public health practice.

In this study, the attributes of children in the T1 (lowest) and T3 (highest) tertiles for all three SSB consumption patterns exhibited distinct characteristics. School-age children, adolescents, and those residing in rural areas were more likely to score higher across all consumption patterns, whereas preschool-age children tended to score lower. These characteristics were consistently observed in the composite SSBs score. Children across different age groups showed varied preferences for types of SSBs. In China, preschoolers from Beijing consumed milk beverages most frequently (63.2%), followed by fruit/vegetable beverages (60.8%), plant-based protein beverages (50.0%), and carbonated beverages (34.2%) [8]. Teenagers from northeastern China consumed tea beverages and carbonated beverages most frequently, followed by sweetened fruit juice and milk beverages [13]. However, all of the aforementioned studies were conducted in limited regions and are therefore unable to reflect the nationwide situation. In this study, we used national data to identify the main SSB consumption patterns among Chinese children and adolescents. Furthermore, we found that children with high scores on the Carbonated Beverage and Milk Tea Pattern had higher odds of overweight/obesity compared with those with low scores among girls. While among boys, higher scores on the Functional Beverage Pattern and Plant Hybrid Pattern were associated with greater odds of overweight or obesity relative to low scores. These findings can help inform the design of targeted interventions involving SSB consumption, which should take consumption preferences and habits into account. With the increasing popularity of sugar-free beverages in recent years, consumption preferences have diverged across sex, with females purchasing more [26]. In the future, SSB consumption patterns are expected to change due to the growing preference for sugar-free alternatives.

We found that children whose family annual income per capita weas in the lowest level, with parents whose education level were none or primary and occupations were unskilled workers or homemakers, and whose nutritional status were overweight or obesity, were more likely to receive a high score across all the patterns. Family factors play vital roles in cultivating and shaping children’s eating habits and further nutritional status. Children from better family economic situations, having higher parental education level and occupation, usually indicated that they had more chance to consume vegetables and fruits, and less possibility to have the habit of drinking SSBs [6,10,11]. Parents with higher education levels may exert health-related knowledge into daily feeding practice, while parents’ ability to make healthy dietary choices may be impacted by financial situation [27,28]. Our findings highlight the importance of considering multiple SES indicators to better capture the complex social determinants involved in dietary intake and childhood obesity risk. These results also suggest that future interventions could take family context and socioeconomic factors into account to identify and target vulnerable populations more effectively.

In recent years, the health condition of children living in rural areas has become an increasing concern. The prevalence of obesity among rural children has risen, with a narrowing gap in obesity rates between urban and rural areas in China, as observed across seven cycles of the Chinese National Surveys on Students’ Constitution and Health [4]. In our study, we found that children from rural areas tended to score higher across all SSB consumption patterns as well as in the composite SSBs score. Furthermore, we also found that living in small and medium-sized cities or rural areas was associated with a higher composite SSBs score than living in large cities. With economic development and rising household income, rural children now have increased access to unhealthy foods, including various types of SSBs [29]. However, both children and their caregivers in rural areas often lack adequate food and nutrition literacy, which is crucial for making healthy dietary choices [30,31]. Previous studies have demonstrated that higher levels of nutrition literacy were inversely associated with overweight/obesity and positively correlated with healthier eating behaviors among adolescents [32,33]. Existing obesity prevention strategies have mainly tended to be urban-centered, leaving a gap in rural-specific health education and interventions. Future efforts should place greater emphasis on addressing the health needs of populations in rural areas.

In this study, all identified SSB consumption patterns were positively associated with a higher likelihood of childhood overweight/obesity. Moreover, children who scored higher in two or more SSB consumption patterns exhibited greater odds of being overweight/obese compared to those with lower scores across all patterns. After further adjustment for quantity of SSB intake in Model 2, the association remained significant. Our study primarily focused on patterns of SSB consumption rather than individual beverage type. The consistent association of all three patterns with greater odds of overweight/obesity indicated that different categories of SSBs may contribute to weight gain through partly shared, yet also distinct biological and behavioral mechanisms. The Carbonated Beverage and Milk Tea Patterns often include energy-dense drinks with high levels of sugar and fat, while the Functional Beverage Pattern may affect metabolic regulation and sleep patterns due to caffeine content [34,35,36]. Furthermore, children with high scores across two or more SSB patterns may reflect a general preference and habitual consumption of SSBs. After adjusting for quantity of SSB intake, the strength of the association between SSB patterns and overweight/obesity remained, indicating that except for SSB intake quantity, other factors such as increased energy intake, sugar addiction, and insulin resistance may also play a role [37]. These findings highlight the complexity of SSB consumption behaviors and suggest potential distinct pathways through which different consumption preferences may exert their effects, offering implications for future research.

This study had several notable strengths. To our knowledge, it was the first to identify major SSB consumption patterns among Chinese children and adolescents using a large, nationally representative sample. It was also the first study to examine the association between different SSB consumption patterns and overweight/obesity in this population. We used principal component factor analysis to derive SSB consumption patterns, providing a more comprehensive interpretation of SSB intake beyond individual beverage types. Furthermore, composite SSBs score was created to capture children’s potential preferences for multiple types of SSBs. The study also accounted for a broad range of sociodemographic and family-related factors, including parental education and occupation, allowing for a more comprehensive assessment of the socioeconomic context shaping SSB consumption and its relationship with childhood overweight/obesity. Additionally, dietary intake was assessed using a non-consecutive 3-day 24 h dietary recall method, which effectively mitigates the problem of high dietary similarity commonly observed in consecutive survey days and provides a more accurate representation of household dietary intake. Furthermore, the large sample size of children ensured adequate statistical power to conduct stratified analyses across subgroups.

However, several limitations should be acknowledged. First, the cross-sectional design precludes the establishment of causal inferences between SSB consumption patterns and childhood overweight/obesity. Nevertheless, this study provided an important snapshot of recent SSB consumption habits among Chinese children and adolescents, offering valuable insights into current public health priorities. Future longitudinal studies incorporating more comprehensive behavioral and lifestyle variables are needed to confirm the observed associations and to elucidate the underlying mechanisms linking different SSB consumption preferences to weight-related outcomes. Second, dietary intake data obtained through non-consecutive 3-day 24 h dietary recall may be subject to recall bias. However, given the large-scale national survey context, along with standardized measurement tools and training, the potential bias was likely minimized. Third, although the factor analysis successfully identified three major SSB consumption patterns among Chinese children, the cumulative variance explained was moderate. This suggests a diversity in consumption habits that may not be fully captured by these patterns. Nonetheless, the identified patterns captured three main consumption patterns among Chinese children and adolescents, providing meaningful insight for developing targeted public health interventions and highlighting the importance of considering the differences in nutritional components among SSB types in future research and policy-making.

## 5. Conclusions

This study identified three major SSB consumption patterns among Chinese children and adolescents and explored the relationship between three patterns and childhood overweight/obesity using a large, nationally representative sample. We found that consumption preferences varied across children and adolescents with different socioeconomic characteristics. After adjusting for covariates, higher scores across all three SSB consumption patterns were positively related to childhood overweight/obesity, particularly among children with high consumption across two or more patterns. The association between the Plant Hybrid Pattern score and overweight/obesity differed significantly between sex. Importantly, tailored health promotion strategies should be developed for SSB consumption habits of children and adolescents.

## Figures and Tables

**Table 1:** Basic characteristics of study participants (*n* = 7979).

Variables	Total (*n* = 7979)	Normal Weight (*n* = 5397)	Overweight (*n* = 1585)	Obesity (*n* = 997)
Age in years, mean ± SD	9.10 ± 3.93	9.45 ± 4.05	8.71 ± 3.70	7.87 ± 3.25
Age in years, median (IQR)	9.0 (6.0)	9.0 (7.0)	9.0 (6.0)	8.0 (5.0)
Age, *n* (%)				
Preschool	2479 (31.1)	1588 (29.4)	514 (32.4)	377 (37.8)
School age	3161 (39.6)	1992 (36.9)	694 (43.8)	475 (47.6)
Adolescent	2339 (29.3)	1817 (33.7)	377 (23.8)	145 (14.5)
Sex, *n* (%)				
Boy	4168 (52.2)	2603 (48.2)	911 (57.5)	654 (65.6)
Girl	3811 (47.8)	2794 (51.8)	674 (42.5)	343 (34.4)
Area, *n* (%)				
Large city	2252 (28.2)	1457 (27.0)	498 (31.4)	297 (29.8)
Small and medium sized city	2584 (32.4)	1768 (32.8)	496 (31.3)	320 (32.1)
Rural	3143 (39.4)	2172 (40.2)	591 (37.3)	380 (38.1)
Father’s educational level, *n* (%)				
None or primary only	714 (8.9)	481 (8.9)	131 (8.3)	102 (10.2)
Secondary	3803 (47.7)	2576 (47.7)	747 (47.1)	480 (48.1)
Trade school/college/university	1888 (23.7)	1261 (23.4)	404 (25.5)	223 (22.4)
NA	1574 (19.7)	1079 (20.0)	303 (19.1)	192 (19.3)
Mother’s educational level, *n* (%)				
None or primary only	1364 (17.1)	950 (17.6)	243 (15.3)	171 (17.2)
Secondary	3964 (49.7)	2662 (49.3)	815 (51.4)	487 (48.8)
Trade school/college/university	1925 (24.1)	1294 (24.0)	387 (24.4)	244 (24.5)
NA	726 (9.1)	491 (9.1)	140 (8.8)	95 (9.5)
Father’s occupation, *n* (%)				
Unskilled worker or homemaker	2791 (35.0)	1902 (35.2)	528 (33.3)	361 (36.2)
Skilled worker	1982 (24.8)	1294 (24.0)	425 (26.8)	263 (26.4)
Professional/manager	1632 (20.5)	1122 (20.8)	329 (20.8)	181 (18.2)
NA	1574 (19.7)	1079 (20.0)	303 (19.1)	192 (19.3)
Mother’s occupation, *n* (%)				
Unskilled worker or homemaker	4297 (53.9)	2931 (54.3)	839 (52.9)	527 (52.9)
Skilled worker	1781 (22.3)	1162 (21.5)	378 (23.8)	241 (24.2)
Professional/manager	1175 (14.7)	813 (15.1)	228 (14.4)	134 (13.4)
NA	726 (9.1)	491 (9.1)	140 (8.8)	95 (9.5)
Family annual income per capita, *n* (%)				
<10,000	2458 (30.81)	1712 (31.7)	441 (27.8)	305 (30.6)
≥10,000–20,000	3057 (38.31)	2074 (38.4)	607 (38.3)	376 (37.7)
≥20,000	2464 (30.88)	1611 (29.8)	537 (33.9)	316 (31.7)
Father’s BMI, mean ± SD	24.30 ± 3.26	24.05 ± 3.16	24.69 ± 3.31	24.97 ± 3.54
Father’s nutritional status, *n* (%)				
Normal weight	3067 (38.4)	2186 (40.5)	559 (35.3)	322 (32.3)
Overweight	2429 (30.4)	1600 (29.6)	194 (12.2)	148 (14.8)
Obesity	770 (9.7)	428 (7.9)	509 (32.1)	320 (32.1)
Underweight	139 (1.7)	104 (1.9)	20 (1.3)	15 (1.5)
NA	1574 (19.7)	1079 (20.0)	303 (19.1)	192 (19.3)
Mother’s BMI, mean ± SD	23.30 ± 3.25	23.02 ± 3.14	23.70 ± 3.28	24.20 ± 3.53
Mother’s nutritional status, *n* (%)				
Normal weight	4248 (58.6)	3018 (55.9)	785 (49.5)	445 (44.6)
Overweight	2076 (28.6)	328 (6.1)	149 (9.4)	127 (12.7)
Obesity	604 (8.3)	1310 (24.3)	459 (29.0)	307 (30.8)
Underweight	325 (4.5)	250 (4.6)	52 (3.3)	23 (2.3)
NA	726 (9.1)	491 (9.1)	140 (8.8)	95 (9.5)

**Table 2:** Characteristics of SSB consumption patterns score tertiles (T) among study participants.

Variable ^a^	Carbonated Beverage and Milk Tea Pattern		*p*	Functional Beverage Pattern		*p*	Plant Hybrid Pattern		*p*
	T1	T3		T1	T3		T1	T3	
Age, *n* (%)			<0.001 *			<0.001 *			<0.001 *
Preschool	1306 (65.79%)	679 (34.21%)		1416 (72.10%)	548 (27.90%)		1268 (64.40%)	701 (35.60%)	
School age	773 (41.03%)	1111 (58.97%)		709 (37.12%)	1201 (62.88%)		734 (38.73%)	1161 (61.27%)	
Adolescent	581 (40.07%)	869 (59.93%)		535 (37.02%)	910 (62.98%)		658 (45.22%)	797 (54.78%)	
Sex, *n* (%)			0.774			0.127			0.060
Boy	1409 (50.21%)	1397 (49.79%)		1370 (49.00%)	1426 (51.00%)		1434 (51.25%)	1364 (48.75%)	
Girl	1251 (49.78%)	1262 (50.22%)		1290 (51.13%)	1233 (48.87%)		1226 (48.63%)	1295 (51.37%)	
Area, *n* (%)			<0.001 *			<0.001 *			<0.001 *
Large city	1455 (73.41%)	527 (26.59%)		1261 (74.84%)	424 (25.16%)		1354 (77.95%)	383 (22.05%)	
Small and medium-sized city	794 (51.33%)	753 (48.67%)		727 (52.26%)	664 (47.74%)		754 (55.85%)	596 (44.15%)	
Rural	411 (22.96%)	1379 (77.04%)		672 (29.96%)	1571 (70.04%)		552 (24.73%)	1680 (75.27%)	
Father’s educational level, *n* (%)			<0.001 *			<0.001 *			<0.001 *
None/primary only	174 (38.84%)	274 (61.16%)		193 (41.51%)	272 (58.49%)		162 (35.14%)	299 (64.86%)	
Secondary	1128 (46.23%)	1312 (53.77%)		1187 (47.54%)	1310 (52.46%)		1165 (46.99%)	1314 (53.01%)	
Trade school/college/university	872 (62.29%)	528 (37.71%)		781 (60.22%)	516 (39.78%)		856 (65.14%)	458 (34.86%)	
Mother’s educational level, *n* (%)			<0.001 *			<0.001 *			<0.001 *
None/primary only	314 (37.97%)	513 (62.03%)		364 (40.99%)	524 (59.01%)		303 (34.24%)	582 (65.76%)	
Secondary	1240 (47.77%)	1356 (52.23%)		1271 (48.05%)	1374 (51.95%)		1265 (48.15%)	1362 (51.85%)	
Trade school/college/university	887 (61.77%)	549 (38.23%)		807 (60.95%)	517 (39.05%)		883 (65.65%)	462 (34.35%)	
Father’s occupation, *n* (%)			<0.001 *			<0.001 *			<0.001 *
Unskilled worker/homemaker	776 (43.89%)	992 (56.11%)		802 (44.33%)	1007 (55.67%)		748 (41.83%)	1040 (58.17%)	
Skilled worker	746 (55.26%)	604 (44.74%)		730 (55.34%)	589 (44.66%)		758 (57.34%)	564 (42.66%)	
Professional/manager	652 (55.73%)	518 (44.27%)		629 (55.61%)	502 (44.39%)		677 (59.18%)	467 (40.82%)	
Mother’s occupation, *n* (%)			<0.001 *			<0.001 *			<0.001 *
Unskilled worker/homemaker	1266 (45.82%)	1497 (54.18%)		1346 (46.90%)	1524 (53.10%)		1268 (44.55%)	1578 (55.45%)	
Skilled worker	687 (56.40%)	531 (43.60%)		662 (56.20%)	516 (43.80%)		682 (57.55%)	503 (42.45%)	
Professional/manager	488 (55.58%)	390 (44.42%)		434 (53.65%)	375 (46.35%)		501 (60.65%)	325 (39.35%)	
Family annual income per capita, *n* (%)			<0.001 *			<0.001 *			<0.001 *
<10,000	770 (45.86%)	909 (54.14%)		760 (46.54%)	873 (53.46%)		728 (44.80%)	897 (55.20%)	
≥10,000–20,000	925 (47.78%)	1011 (52.22%)		977 (49.22%)	1008 (50.78%)		929 (47.13%)	1042 (52.87%)	
≥20,000	965 (56.63%)	739 (43.37%)		923 (54.26%)	778 (45.74%)		1003 (58.21%)	720 (41.79%)	
Father’s nutritional status, *n* (%)			0.155			0.575			0.480
Normal weight	1029 (49.85%)	1035 (50.15%)		1052 (51.75%)	981 (48.25%)		1033 (50.69%)	1005 (49.31%)	
Overweight	854 (52.65%)	768 (47.35%)		808 (50.19%)	802 (49.81%)		832 (51.87%)	772 (48.13%)	
Obesity	246 (47.58%)	271 (52.42%)		256 (49.14%)	265 (50.86%)		262 (50.78%)	254 (49.22%)	
Underweight	45 (52.94%)	40 (47.06%)		45 (47.37%)	50 (52.63%)		56 (58.33%)	40 (41.67%)	
Mother’s nutritional status, *n* (%)			<0.001 *			0.005 *			<0.001 *
Normal weight	1447 (51.31%)	1373 (48.69%)		1410 (49.89%)	1416 (50.11%)		1465 (51.71%)	1368 (48.29%)	
Overweight	662 (47.46%)	733 (52.54%)		689 (49.36%)	707 (50.64%)		654 (47.08%)	735 (52.92%)	
Obesity	191 (46.47%)	220 (53.53%)		208 (49.76%)	210 (50.24%)		200 (48.19%)	215 (51.81%)	
Underweight	141 (60.52%)	92 (39.48%)		135 (62.21%)	82 (37.79%)		132 (60.00%)	88 (40.00%)	

^a^ The categorical variables are displayed as counts (%) in the dataset. The chi-square test was employed. * indicates a significant difference at *p* < 0.05.

**Table 3:** Odds ratios for the association between three SSB consumption patterns score and childhood overweight/obesity.

Variable	Model 1 ^a^			Model 2 ^b^		
	OR	95% CI	*p*	OR	95% CI	*p*
Carbonated Beverage and Milk Tea Pattern						
Low ^c^	ref			ref		
High	1.151	1.010, 1.312	0.035 *	1.158	1.015, 1.321	0.029 *
Functional Beverage Pattern						
Low	ref			ref		
High	1.216	1.071, 1.381	0.003 *	1.216	1.071, 1.381	0.003 *
Plant Hybrid Pattern						
Low	ref			ref		
High	1.164	1.028, 1.320	0.017 *	1.162	1.026, 1.317	0.018 *
Composite SSBs score						
Low ^d^	ref			ref		
Low-medium	1.234	0.873, 1.746	0.234	1.261	0.890, 1.787	0.192
Medium-high	1.236	1.043, 1.465	0.014 *	1.249	1.053, 1.482	0.011 *
High	1.253	1.079, 1.456	0.003 *	1.256	1.081, 1.459	0.003 *
	*p* for trend = 0.004 *			*p* for trend = 0.003 *		

^a^ Adjusted for age in years, sex, area, parental education level, mother’s BMI, protein energy ratio, family annual income per capita, and household clustering as a random effect term. ^b^ Further adjusted for quantity of SSB intake ≥ 300 mL/day. ^c^ The reference group was designated as Low. ^d^ Low indicates that all three SSB consumption pattern scores were low; Low-medium indicates that only one pattern score was high; Medium-high indicates that two pattern scores were high; and High indicates that all three pattern scores were high. Abbreviation: OR = odds ratio; CI = confidence interval. * indicates a significant difference at *p* < 0.05.

## Data Availability

The original contributions presented in this study are included in the article. Further inquiries can be directed to the corresponding author.

## Supplementary Materials

The following supporting information can be downloaded at https://www.mdpi.com/article/10.3390/nu17213442/s1. Table S1: Factor loadings and SSB consumption patterns for nine types; Table S2: Characteristics of SSB consumption patterns score across tertiles (T1–T3) among study participants; Table S3: Characteristics of composite SSBs score among study participants; Table S4: Odds ratios for the association between three SSB consumption patterns score and childhood overweight; Table S5: Odds ratios for the association between three SSB consumption patterns score and childhood obesity; Table S6: Odds ratios for the association between SSB consumption patterns score and childhood overweight/obesity, subgroup analysis by age; Table S7: Odds ratios for the association between SSB consumption patterns score and childhood overweight/obesity, subgroup analysis by sex; Table S8: Odds ratios for the association between SSB consumption patterns score and childhood overweight/obesity, subgroup analysis by area.

## Abbreviations

The following abbreviations are used in this manuscript:

BMI	body mass index
CIs	confidence intervals
CFCS	Chinese Food Consumption Survey
IQR	interquartile range
KMO	Kaiser-Meyer-Olkin
LRTs	likelihood ratio tests
OR	odds ratio
PPS	Probability Proportional to Size
SD	standard deviation
SSBs	sugar-sweetened beverages
WHO	World Health Organization
