# Supplementary material for: The Association of Sugar-Sweetened Beverages Consumption Patterns and Overweight/Obesity: Evidence from a Large-Scale Survey of Chinese Children and Adolescents
Source: Nutrients. 2025 Oct 31;17(21):3442. doi: 10.3390/nu17213442 (PMC12608719; doi:10.3390/nu17213442)
Supplement: Supplementary file 1 [file nutrients-17-03442-s001.zip › nutrients-3937384-supplementary.pdf]

Supplementary Table S1 Factor loadings and SSB consumption patterns for nine types

SSB Types	Carbonated Beverage and Milk Tea Pattern (Pattern 1)	Functional Beverage Pattern (Pattern 2)	Plant Hybrid Pattern (Pattern 3)
Carbonated beverages	0.546		
Fruit and vegetable juices and their beverages [a]			
Plant protein beverages			0.551
Dairy containing beverages			
Tea (and its types) beverages			
Milk tea beverages	0.669		
Coffee beverages		0.712	
Plant-based beverages			0.641
Sports beverages		0.725	

[a] Factor loadings below an absolute value of 0.5 were excluded for simplification purposes.

Supplementary Table S2 Characteristics of SSB consumption patterns score across tertiles (T1-T3) among study participants

**Variable** [a]	**Carbonated Beverage and Milk Tea Pattern**			**Functional Beverage Pattern**			**Plant Hybrid Pattern**		
	T1	T2	T3	T1	T2	T3	T1	T2	T3
**Age, *n* (%)**									
Preschool	1306 (52.68%)	494 (19.93%)	679 (27.39%)	1416 (57.12%)	515 (20.77%)	548 (22.11%)	1268 (51.15%)	510 (20.57%)	701 (28.28%)
School age	773 (24.45%)	1277 (40.40%)	1111 (35.15%)	709 (22.43%)	1251 (39.58%)	1201 (37.99%)	734 (23.22%)	1266 (40.05%)	1161 (36.73%)
Adolescent	581 (24.84%)	889 (38.01%)	869 (37.15%)	535 (22.87%)	894 (38.22%)	910 (38.91%)	658 (28.13%)	884 (37.79%)	797 (34.07%)
**Sex, *n* (%)**									
Boy	1409 (33.81%)	1362 (32.68%)	1397 (33.52%)	1370 (32.87%)	1372 (32.92%)	1426 (34.21%)	1434 (34.40%)	1370 (32.87%)	1364 (32.73%)
Girl	1251 (32.83%)	1298 (34.06%)	1262 (33.11%)	1290 (33.85%)	1288 (33.80%)	1233 (32.35%)	1226 (32.17%)	1290 (33.85%)	1295 (33.98%)
**Area, *n* (%)**									
Large city	1455 (64.61%)	270 (11.99%)	527 (23.40%)	1261 (55.99%)	567 (25.18%)	424 (18.83%)	1354 (60.12%)	515 (22.87%)	383 (17.01%)
Small and medium-sized city	794 (30.73%)	1037 (40.13%)	753 (29.14%)	727 (28.13%)	1193 (46.17%)	664 (25.70%)	754 (29.18%)	1234 (47.76%)	596 (23.07%)
Rural	411 (13.08%)	1353 (43.05%)	1379 (43.88%)	672 (21.38%)	900 (28.64%)	1571 (49.98%)	552 (17.56%)	911 (28.99%)	1680 (53.45%)

**Father's educational level, *n* (%)**									
None or primary only	174 (24.37%)	266 (37.25%)	274 (38.38%)	193 (27.03%)	249 (34.87%)	272 (38.10%)	162 (22.69%)	253 (35.43%)	299 (41.88%)
Secondary	1128 (29.66%)	1363 (35.84%)	1312 (34.50%)	1187 (31.21%)	1306 (34.34%)	1310 (34.45%)	1165 (30.63%)	1324 (34.81%)	1314 (34.55%)
Trade school/ college/university	872 (46.19%)	488 (25.85%)	528 (27.97%)	781 (41.37%)	591 (31.30%)	516 (27.33%)	856 (45.34%)	574 (30.40%)	458 (24.26%)
**Mother's educational level, *n* (%)**									
None or primary only	314 (23.02%)	537 (39.37%)	513 (37.61%)	364 (26.69%)	476 (34.90%)	524 (38.42%)	303 (22.21%)	479 (35.12%)	582 (42.67%)
Secondary	1240 (31.28%)	1368 (34.51%)	1356 (34.21%)	1271 (32.06%)	1319 (33.27%)	1374 (34.66%)	1265 (31.91%)	1337 (33.73%)	1362 (34.36%)
Trade school/ college/university	887 (46.08%)	489 (25.40%)	549 (28.52%)	807 (41.92%)	601 (31.22%)	517 (26.86%)	883 (45.87%)	580 (30.13%)	462 (24.00%)
**Father's occupation, *n* (%)**									
Unskilled worker or homemaker	776 (27.80%)	1023 (36.65%)	992 (35.54%)	802 (28.74%)	982 (35.18%)	1007 (36.08%)	748 (26.80%)	1003 (35.94%)	1040 (37.26%)
Skilled worker	746 (37.64%)	632 (31.89%)	604 (30.47%)	730 (36.83%)	663 (33.45%)	589 (29.72%)	758 (38.24%)	660 (33.30%)	564 (28.46%)
Professional/manager	652 (39.95%)	462 (28.31%)	518 (31.74%)	629 (38.54%)	501 (30.70%)	502 (30.76%)	677 (41.48%)	488 (29.90%)	467 (28.62%)

**Mother's occupation, *n* (%)**									
Unskilled worker or homemaker	1266 (29.46%)	1534 (35.70%)	1497 (34.84%)	1346 (31.32%)	1427 (33.21%)	1524 (35.47%)	1268 (29.51%)	1451 (33.77%)	1578 (36.72%)
Skilled worker	687 (38.57%)	563 (31.61%)	531 (29.81%)	662 (37.17%)	603 (33.86%)	516 (28.97%)	682 (38.29%)	596 (33.46%)	503 (28.24%)
Professional/manager	488 (41.53%)	297 (25.28%)	390 (33.19%)	434 (36.94%)	366 (31.15%)	375 (31.91%)	501 (42.64%)	349 (29.70%)	325 (27.66%)
**Family annual income per capita, *n* (%)**									
<10,000	770 (31.33%)	779 (31.69%)	909 (36.98%)	760 (30.92%)	825 (33.56%)	873 (35.52%)	728 (29.62%)	833 (33.89%)	897 (36.49%)
≥10,000–20,000	925 (30.26%)	1121 (36.67%)	1011 (33.07%)	977 (31.96%)	1072 (35.07%)	1008 (32.97%)	929 (30.39%)	1086 (35.53%)	1042 (34.09%)
≥20,000	965 (39.16%)	760 (30.84%)	739 (29.99%)	923 (37.46%)	763 (30.97%)	778 (31.57%)	1003 (40.71%)	741 (30.07%)	720 (29.22%)
**Father's nutritional status, *n* (%)**									
Normal weight	1029 (33.55%)	1003 (32.70%)	1035 (33.75%)	1052 (34.30%)	1034 (33.71%)	981 (31.99%)	1033 (33.68%)	1029 (33.55%)	1005 (32.77%)
Overweight	854 (35.16%)	807 (33.22%)	768 (31.62%)	808 (33.26%)	819 (33.72%)	802 (33.02%)	832 (34.25%)	825 (33.96%)	772 (31.78%)
Obesity	246 (31.95%)	253 (32.86%)	271 (35.19%)	256 (33.25%)	249 (32.34%)	265 (34.42%)	262 (34.03%)	254 (32.99%)	254 (32.99%)

Underweight	45 (32.37%)	54 (38.85%)	40 (28.78%)	45 (32.37%)	44 (31.65%)	50 (35.97%)	56 (40.29%)	43 (30.94%)	40 (28.78%)
**Mother's nutritional status, *n* (%)**									
Normal weight	1447 (34.06%)	1428 (33.62%)	1373 (32.32%)	1410 (33.19%)	1422 (33.47%)	1416 (33.33%)	1465 (34.49%)	1415 (33.31%)	1368 (32.20%)
Overweight	662 (31.89%)	681 (32.80%)	733 (35.31%)	689 (33.19%)	680 (32.76%)	707 (34.06%)	654 (31.50%)	687 (33.09%)	735 (35.40%)
Obesity	191 (31.62%)	193 (31.95%)	220 (36.42%)	208 (34.44%)	186 (30.79%)	210 (34.77%)	200 (33.11%)	189 (31.29%)	215 (35.60%)
Underweight	141 (43.38%)	92 (28.31%)	92 (28.31%)	135 (41.54%)	108 (33.23%)	82 (25.23%)	132 (40.62%)	105 (32.31%)	88 (27.08%)

[a] The categorical variables are displayed as counts (%) in the dataset.

Supplementary Table S3 Characteristics of composite SSBs score among study participants

Variable [a]	Composite SSBs score			
	Low	Low-medium	Medium-high	High
**Age, *n* (%)**				
Preschool	1176 (58.36%)	33 (15%)	396 (26.49%)	874 (20.57%)
School age	476 (23.62%)	86 (39.09%)	616 (41.2%)	1983 (46.67%)
Adolescent	363 (18.01%)	101 (45.91%)	483 (32.31%)	1392 (32.76%)
**Sex, *n* (%)**				
Boy	1050 (52.11%)	124 (56.36%)	815 (54.52%)	2179 (51.28%)
Girl	965 (47.89%)	96 (43.64%)	680 (45.48%)	2070 (48.72%)
**Area, *n* (%)**				
Large city	1121 (55.63%)	100 (45.45%)	507 (33.91%)	524 (12.33%)
Small and medium-sized city	526 (26.1%)	71 (32.27%)	555 (37.12%)	1432 (33.7%)
Rural	368 (18.26%)	49 (22.27%)	433 (28.96%)	2293 (53.97%)
**Father's educational level, *n* (%)**				
None or primary only	127 (7.66%)	13 (7.47%)	122 (10.23%)	452 (13.38%)
Secondary	863 (52.02%)	88 (50.57%)	715 (59.93%)	2137 (63.24%)
Trade school/ college/university	669 (40.33%)	73 (41.95%)	356 (29.84%)	790 (23.38%)
**Mother's educational level, *n* (%)**				
None or primary only	230 (12.37%)	19 (9.45%)	253 (18.71%)	862 (22.45%)
Secondary	951 (51.13%)	106 (52.74%)	711 (52.59%)	2196 (57.19%)
Trade school/ college/university	679 (36.51%)	76 (37.81%)	388 (28.7%)	782 (20.36%)
**Father's occupation, *n* (%)**				
Unskilled worker or homemaker	587 (35.38%)	33 (18.97%)	499 (41.83%)	1672 (49.48%)

Skilled worker	565 (34.06%)	70 (40.23%)	399 (33.45%)	948 (28.06%)
Professional/manager	507 (30.56%)	71 (40.8%)	295 (24.73%)	759 (22.46%)
**Mother's occupation, *n* (%)**				
Unskilled worker or homemaker	991 (53.28%)	83 (41.29%)	741 (54.81%)	2482 (64.64%)
Skilled worker	509 (27.37%)	65 (32.34%)	374 (27.66%)	833 (21.69%)
Professional/manager	360 (19.35%)	53 (26.37%)	237 (17.53%)	525 (13.67%)
**Family annual income per capita, *n* (%)**				
<10,000	566 (28.09%)	53 (24.09%)	454 (30.37%)	1385 (32.6%)
≥10,000–20,000	715 (35.48%)	63 (28.64%)	560 (37.46%)	1719 (40.46%)
≥20,000	734 (36.43%)	104 (47.27%)	481 (32.17%)	1145 (26.95%)
**Father's nutritional status, *n* (%)**				
Normal weight	785 (47.32%)	77 (44.25%)	605 (50.71%)	1600 (47.35%)
Overweight	194 (11.69%)	25 (14.37%)	132 (11.06%)	419 (12.4%)
Obesity	643 (38.76%)	65 (37.36%)	435 (36.46%)	1286 (38.06%)
Underweight	37 (2.23%)	7 (4.02%)	21 (1.76%)	74 (2.19%)
**Mother's nutritional status, *n* (%)**				
Normal weight	1093 (58.76%)	119 (59.2%)	805 (59.54%)	2231 (58.1%)
Overweight	157 (8.44%)	15 (7.46%)	98 (7.25%)	334 (8.7%)
Obesity	504 (27.1%)	54 (26.87%)	385 (28.48%)	1133 (29.51%)
Underweight	106 (5.7%)	13 (6.47%)	64 (4.73%)	142 (3.7%)

[a] The categorical variables are displayed as counts (%) in the dataset.

Supplementary Table S4 Odds ratios for the association between three SSB consumption patterns score and childhood overweight

Variable	Model 1 [a]			Model 2 [b]		
	**OR**	**95% CI**	***p***	**OR**	**95% CI**	***p***
Carbonated Beverage and Milk Tea Pattern						
Low [c]	ref			ref		
High	1.157	0.993, 1.349	0.062	1.167	1.001, 1.361	0.049 *
Functional Beverage Pattern						
Low	ref			ref		
High	1.152	0.993, 1.335	0.061	1.153	0.994, 1.336	0.060
Plant Hybrid Pattern						
Low	ref			ref		
High	1.135	0.981, 1.314	0.089	1.132	0.978, 1.311	0.096
Composite SSBs score						
Low [d]	ref			ref		
Low-medium	1.121	0.746, 1.685	0.583	1.155	0.767, 1.740	0.490
Medium-high	1.234	1.012, 1.504	0.038 *	1.252	1.026, 1.528	0.027 *
High	1.203	1.010, 1.434	0.038 *	1.208	1.014, 1.439	0.035 *
	p for trend = 0.036 *			*p* for trend = 0.034 *		

[a] Adjusted for age in years, sex, area, parental education level, mother's BMI, protein energy ratio, family annual income per capita, and household clustering as a random effect term. [b] Further adjusted for quantity of SSB intake ⩾ 300 ml/day. [c] The reference group was designated as Low. [d] Low indicates that all three SSB consumption pattern scores were low; Low-medium indicates that only one pattern score was high; Medium-high indicates that two pattern scores were high; and High indicates that all three pattern scores were high. Abbreviation: OR = odds ratio; CI = confidence interval. * indicates a significant difference at $p < 0.05$.

Supplementary Table S5 Odds ratios for the association between three SSB consumption patterns score and childhood obesity

Variable	Model 1 [a]			Model 2 [b]		
	OR	95% CI	*p*	OR	95% CI	*p*
Carbonated Beverage and Milk Tea Pattern						
Low [c]	ref			ref		
High	1.771	0.866, 3.622	0.118	1.710	0.832, 3.513	0.145
Functional Beverage Pattern						
Low	ref			ref		
High	2.353	1.152, 4.807	0.019 *	2.328	1.139, 4.760	0.021 *
Plant Hybrid Pattern						
Low	ref			ref		
High	1.783	0.893, 3.562	0.101	1.798	0.900, 3.594	0.097
Composite SSBs score						
Low [d]	ref			ref		
Low-medium	1.140	0.076, 16.991	0.925	0.919	0.059, 14.271	0.952
Medium-high	2.528	1.006, 6.353	0.049 *	2.384	0.939, 6.053	0.068
High	2.235	0.996, 5.013	0.051	2.213	0.985, 4.973	0.055
	p for trend = 0.038 *			*p* for trend = 0.042 *		

[a] Adjusted for age in years, sex, area, parental education level, mother's BMI, protein energy ratio, family annual income per capita, and household clustering as a random effect term. [b] Further adjusted for quantity of SSB intake ⩾ 300 ml/day. [c] The reference group was designated as Low. [d] Low indicates that all three SSB consumption pattern scores were low; Low-medium indicates that only one pattern score was high; Medium-high indicates that two pattern scores were high; and High indicates that all three pattern scores were high. Abbreviation: OR = odds ratio; CI = confidence interval. * indicates a significant difference at $p < 0.05$.

Supplementary Table S6 Odds ratios for the association between SSB consumption patterns score and childhood overweight/obesity, subgroup analysis by age

Variable	Preschool [a]			School age			Adolescent		
	OR	95% CI	*p*	OR	95% CI	*p*	OR	95% CI	*p*
**Carbonated Beverage and Milk Tea Pattern**									
Low [b]	ref			ref			ref		
High	1.221	0.985, 1.514	0.068	0.953	0.761, 1.193	0.673	1.260	0.901, 1.760	0.176
p for Pattern 1 * Age = 0.718									
**Functional Beverage Pattern**									
Low	ref			ref			ref		
High	1.058	0.841, 1.331	0.628	1.243	1.003, 1.540	0.047 *	1.243	0.906, 1.707	0.178
p for Pattern 2 * Age = 0.210									
**Plant Hybrid Pattern**									
Low	ref			ref			ref		
High	1.194	0.957, 1.491	0.116	1.058	0.851, 1.315	0.611	1.148	0.853, 1.545	0.361
p for Pattern 3 * Age = 0.995									

[a] Adjusted for age in years, sex, area, parental education level, mother's BMI, protein energy ratio, family annual income per capita, quantity of SSB intake ⩾ 300 ml/day, and household clustering as a random effect term. [b] The reference group was designated as Low. Abbreviation: OR = odds ratio; CI = confidence interval. * indicates a significant difference at $p < 0.05$.

Supplementary Table S7 Odds ratios for the association between SSB consumption patterns score and childhood overweight/obesity, subgroup analysis by sex

Variable	Boy [a]			Girl		
	OR	95% CI	*p*	OR	95% CI	*p*
**Carbonated Beverage and Milk Tea Pattern**						
Low [b]	ref			ref		
High	1.114	0.942, 1.316	0.207	1.234	1.010, 1.508	0.040 *
p for Pattern 1 * Sex = 0.562						
**Functional Beverage Pattern**						
Low	ref			ref		
High	1.295	1.098, 1.526	0.002 *	1.109	0.919, 1.339	0.281
p for Pattern 2 * Sex = 0.005 *						
**Plant Hybrid Pattern**						
Low	ref			ref		
High	1.184	1.011, 1.387	0.037 *	1.146	0.946, 1.389	0.163
p for Pattern 3 * Sex = 0.165						

[a] Adjusted for age in years, area, parental education level, mother's BMI, protein energy ratio, family annual income per capita, quantity of SSB intake ⩾ 300 ml/day, and household clustering as a random effect term. [b] The reference group was designated as Low. Abbreviation: OR = odds ratio; CI = confidence interval. * indicates a significant difference at $p < 0.05$.

Supplementary Table S8 Odds ratios for the association between SSB consumption patterns score and childhood overweight/obesity, subgroup analysis by area

Variable	Large city [a]			Small and medium-sized city			Rural area		
	OR	95% CI	*p*	OR	95% CI	*p*	OR	95% CI	*p*
**Carbonated Beverage and Milk Tea Pattern**									
Low [b]	ref			ref			ref		
High	1.082	0.879, 1.331	0.458	1.181	0.958, 1.455	0.119	1.270	0.948, 1.700	0.109
p for Pattern 1 * Area = 0.793									
**Functional Beverage Pattern**									
Low	ref			ref			ref		
High	1.147	0.938, 1.401	0.181	1.287	1.031, 1.605	0.025 *	1.258	0.982, 1.612	0.070
p for Pattern 2 * Area = 0.502									
**Plant Hybrid Pattern**									
Low	ref			ref			ref		
High	1.134	0.930, 1.382	0.214	1.192	0.965, 1.472	0.103	1.203	0.936, 1.546	0.149
p for Pattern 3 * Area = 0.833									

[a] Adjusted for age in years, sex, parental education level, mother's BMI, protein energy ratio, family annual income per capita, quantity of SSB intake ⩾ 300 ml/day, and household clustering as a random effect term. [b] The reference group was designated as Low. Abbreviation: OR = odds ratio; CI = confidence interval. * indicates a significant difference at $p < 0.05$.
